# Planning and evaluating health programs: contributions of the RE-AIM framework to Nursing

**DOI:** 10.1590/0104-1169.0000.2447

**Published:** 2014

**Authors:** Fabio Araujo Almeida, Fabiana Almeida Brito

**Affiliations:** Director of the Implementation and Systems Science Laboratory at Virginia Tech. Assistant Professor, Department of Human Nutrition, Foods, and Exercise, Faculty of Health Sciences at Virginia Tech, Blacksburg, VA, USA. E-mail: falmeida@vt.edu; Doctoral student in the Department of Human Nutrition, Foods, and Exercise at Virginia Tech, Blacksburg, VA, USA, fabiana@vt.edu

There is little doubt that health is one of the most basic necessities of the individual^(^ 1 ^)^ Nevertheless, the prevalence of preventable diseases continue to grow globally. In fact, preventable diseases such as cardiovascular disease, diabetes, obesity, and some types of cancer already kill more people than any other cause^(^ 2 ^)^. As a result over the previous four decades there has been an incredible amount of research that tests the efficacy of different interventions and treatments to help prevent and or treat preventable chronic diseases, and much has been learned^(^ 3 ^)^. Still, there is little evidence that this knowledge is being translated into typical clinical or community practice^(^ 3 ^)^.

To address this issue, Glasgow et al.^(^ 4 ^)^ suggested that the success of health programs should be evaluated based on both individual and organizational factors. In that seminal article they introduced the RE-AIM framework (Reach, Effectiveness, Adoption, Implementation, Maintenance) as a method to balance the focus of research on both internal and external validity and it included outcomes that are operationalized at the individual level, the organizational level, or both.

At the individual level, reach and effectiveness are assessed based upon the individuals who participate and are ultimately intended to receive a health benefit from a program^(^ 4 ^)^. Reach is defined as the number of participants, proportion of the target population that participates, and the representativeness of participants to the target population. Effectiveness is assessed as changes in the primary outcome, potential negative outcomes, and quality of life.

At the organizational level adoption and implementation are assessed based upon the delivery setting or delivery staff level^(^ 4 ^)^. Adoption is defined in terms of settings or delivery staff that would implement and sustain a given intervention. Thus, adoption is the number of settings/staff, proportion of the target population of settings/staff that participates, and the representativeness of participating settings/staff. Implementation includes the degree to which an intervention is delivered as intended and the costs associated with the intervention. Finally, maintenance can be defined as the degree to which an effect is sustained at the individual level at least 6 months post program. It is also defined as the degree to which an intervention can be sustained after the formal research funding is completed.

Since its original introduction, the RE-AIM framework has been expanded and used in a variety of fields including aging^(^ 5 ^)^, cancer screening^(^ 5 ^)^, dietary change^(^ 5 ^)^, physical activity^(^ 5 ^)^, medication adherence^(^ 5 ^)^, public health policy^(^ 5 ^)^, chronic illness self-management^(^ 5 ^)^, women's health^(^ 5 ^)^, HIV^(^ 5 ^)^, smoking cessation^(^ 5 ^)^, diabetes prevention^(^ 5 ^)^ and many others. The framework has been used in systematic reviews of the literature^(^ 5 ^)^ as well as a guiding tool in the planning and evaluation of health programs and policies^(^ 5 ^)^. More recently, after growing interest from Brazilian researchers and public health administrators, the framework was translated and culturally adapted to the Brazilian reality in 2013^(^ 6 ^)^.

The RE-AIM framework presents a unique tool for the nursing field, since ever more often the nursing profession is engaged in the development, implementation and evaluation of health programs and policies. Using RE-AIM as a guide and recognizing its theoretical and practical consistencies, nurses can plan and be active members in the construction of new interventions and programs in the various levels of public and private health care.

Furthermore, the evaluation of health programs, which are often led by nurses, is a central component in improving the quality of health care. This is a field that nurses have much to contribute to its advancement. Future advances will depend on the systematic evaluation and dissemination of successful practices. Thus, the RE-AIM framework can serve as a tool to the nursing profession in the planning and evaluation of health programs that reach a large and representative sample of the target population with effective strategies that are easily adopted, implemented, and sustained over time by different settings leading to better quality health care, patient outcomes, and a greater public health impact.
